# Applications and Restrictions of Integrated Genomic and Metabolomic Screening: An Accelerator for Drug Discovery from Actinomycetes?

**DOI:** 10.3390/molecules26185450

**Published:** 2021-09-07

**Authors:** Janina Krause

**Affiliations:** Abteilung Biomedizinische Grundlagen 1, Institut für Gesundheitsforschung und Bildung, Universität Osnabrück, 49076 Osnabrück, Germany; janina.krause@uni-osnabrueck.de

**Keywords:** bioactive natural products, actinomycetes, genome mining, biosynthetic gene cluster, dereplication, molecular networking

## Abstract

Since the golden age of antibiotics in the 1950s and 1960s actinomycetes have been the most prolific source for bioactive natural products. However, the number of discoveries of new bioactive compounds decreases since decades. New procedures (e.g., activating strategies or innovative fermentation techniques) were developed to enhance the productivity of actinomycetes. Nevertheless, compound identification remains challenging among others due to high rediscovery rates. Rapid and cheap genome sequencing as well as the advent of bioinformatical analysis tools for biosynthetic gene cluster identification in combination with mass spectrometry-based molecular networking facilitated the tedious process of dereplication. In recent years several studies have been dedicated to accessing the biosynthetic potential of *Actinomyces* species, especially streptomycetes, by using integrated genomic and metabolomic screening in order to boost the discovery rate of new antibiotics. This review aims to present the various possible applications of this approach as well as the newly discovered molecules, covering studies between 2014 and 2021. Finally, the effectiveness of this approach with regard to find new bioactive agents from actinomycetes will be evaluated.

## 1. Introduction

Ever since Alexander Fleming discovered the ability of the fungus *Penicillium rubens (notatum*) to produce an antibiotic substance in 1929, microbes have been a rich source for bioactive molecules [1]. Among microbes actinomycetes are the most productive order known: e.g., between 1940 and 1970, 70% of all antibiotics or derivatives thereof have been isolated from actinomycetes [2]. Besides antibiotics, natural products with antiprolific, immunosuppressant, herbicidal and antiviral activity have been isolated from actinomycetes [3,4].

While early searches for bioactive agents were based simply on testing the bioactive capacities of raw culture extracts on diffusion-assays [5], more elaborated methods, like target-based approaches and high-throughput screening, had to be applied when the rediscovery rate of already described natural products increased [6], At the beginning of the century, the search for new bioactive substances was boosted by the possibility of relatively cheap whole-genome sequencing with next-generation sequencing techniques and the development of bioinformatic methods for genome mining [7], To identify the genes, which are relevant for the synthesis of a secondary metabolite, it comes in handy that these genes generally accumulate in one contiguous region in the genome called biosynthetic gene cluster (BGC) [8]. 

**Figure 1:**
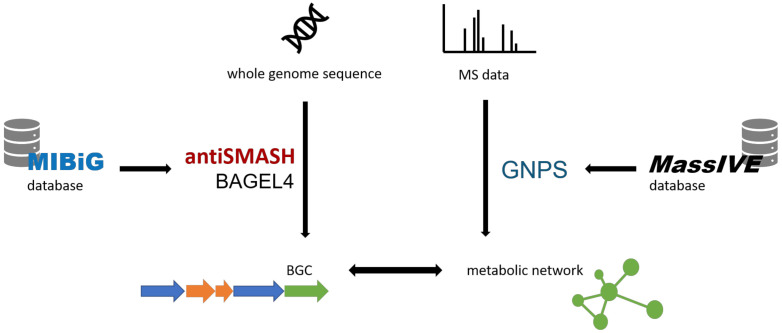
Overview over the bioinformatic tools involved in genomic and metabolomic screening, On the one hand, antiSMASH [10,11] and BAGEL4 [12,13] process the genome sequence to identify BGCs resorting to the MIBiG database [14]. On the other hand, MS data are converted into a molecular network by GNPS [15], which accesses deposited MS data from the MassIVE database [16].

In recent years, genome mining has been a main driver of natural product discovery [9]. Many bioinformatic tools have been developed to access the biosynthetic potential of microbes. Several of those tools will be referred to in the Section 2. These shall be introduced in the following (Figure 1). 

### 1.1. Bioinformatic Tools for Genome Mining

Several algorithms exist to screen whole genomes for BGCs. The most frequently used Webtool is the Antibiotics and Secondary Metabolites Analysis Shell (antiSMASH) [10,11] which was developed to integrate all existing methods for the identification of BGCs in one user friendly platform. The distinct regions identified in the genome are aligned to their nearest relatives deposited in the antiSMASH database. A forecast about the number of BGCs in the whole genome, the BGC’s borders and the presumed class of secondary metabolite is given. Also, the similarity to identified clusters is displayed [17].

Similar to antiSMASH, the Bacteriocin Genome mining tooL (BAGEL) serves to identify BGCs but with focus on Ribosomally synthesized and Post translationally modified Peptides (RiPPs) and bacteriocins. The whole genome in question is screened via comparison against a core peptide database [12,13].

The applicability of cluster finding tools like antiSMASH and BAGEL depends on the quality and quantity of the available genome data. These tools’ databases revert to the Minimum Information about a Biosynthetic Gene Cluster (MIBiG) database, which is a manually curated repository for genome data. For standardization an own data standard has been developed [14].

### 1.2. Bioinformatic Tools for Metabolomic Analysis

The analysis of genome sequencing data demonstrates that on average about 20 secondary metabolites are encoded in the genome of actinomycetes [8], while only about 10% of these are actually expressed [18]. But the genome by itself does not give information about the metabolites that are actually produced by a strain. To investigate the metabolome, tandem mass spectroscopy has been established as the method of choice [19]. By comparison of the metabolites’ spectra to spectral libraries, known compounds can be identified early in the process of dereplication and thus the tedious process of isolation and structure elucidation is avoided. The detected chemical entities can be visualized in a so-called molecular network. A molecular network displays the relations or similarities, respectively, between the spectra of related/similar molecules. The spectra are depicted as so called ‘nodes’ or ‘parent ions’, which are connected by ‘edges’, to visualize alignments between nodes. Prerequisite for this method is a common platform on which MS/MS-data of preferably all known substances are stored and can be accessed for comparison with one’s own data set [15]. 

The platform Global Natural Products Social Molecular Networking (GNPS) enables storage, analysis and sharing of MS/MS-data for molecular networking. Via GNPS-clustering MS/MS-data can be matched with the GNPS-database, which is built on user-data [15]. The Mass spectrometry Interactive Virtual Environment (MassIVE) database is the public repository of GNPS for MS/MS-data [16]. 

Other bioinformatic tools rely on the data processed by GNPS. DEREPLICATOR started as an identifier for peptidic natural products but was soon extended for other classes of natural products. Similar to GNPS, the aim of this tool is to identify known natural products before traversing an immense dereplication effort via compound isolation and structure elucidation. For this purpose, DEREPLICATOR accesses the large spectral library of GNPS [20]. DEREPLICATOR however requires at least one unmodified peptide within a node of a family of molecules as only spectral networks can be identified. To overcome this drawback, VarQuest was developed. VarQuest calculates the masses of mutated peptide variants from a peptidic database and matches these variants against recorded spectra to identify the best fit. The VarQuest algorithm’s major advantage is to efficiently narrowing down the number of candidate peptidic variants, which accelerates the calculation immensely [21]. 

The approaches to study the genome as well as the metabolome have been successfully joined in an integrated genomic and metabolomic screening, also called metabologenomics [22] or comparative genomics and metabolomics [23]. With this method, the detected parent ions in a molecular network are linked to the corresponding biosynthetic pathway, derived from the BGC [24]. That offers the possibility to derive details about structural features, which cannot be derived from the mass-spectrum exclusively, like stereochemistry [25], or to identify targets for genetic engineering [26].

This review aims to highlight studies, in which integrated genomic and metabolomic screening was performed on actinomycetes, and to elaborate their various possible applications. Also, an overview over newly discovered molecules from actinomycetes detected by this method is provided. Finally, the review will demonstrate the usefulness of this method for the above-mentioned applications but rather not for the discovery of new chemical bioactive scaffolds. 

## 2. Applications of Genomic and Metabolomic Screening

### 2.1. Discovery of New Analogs

Integrated genomic and metabolomic screening is a powerful tool for the detection of new derivatives of known compounds.

Liu et al. [27] investigated the genome and metabolome of the known daptomycin-producer *Streptomyces roseosporus* in search for nonribosomal peptide (NRP) antibiotics. They searched for specific peptidic signatures, that is masses of amino acid fragments, in the molecular network of the strain’s metabolome. This way, they discovered subnodes of the daptomycin cluster, which correspond to daptomycin variants missing the N-terminal lipid chain and tryptophan. Additionally, sodium- and potassium- adducts of arylomycin [27], a lipohexapeptide with antibiotic activity [28], were discovered. The authors especially emphasized the existence of several arylomycin intermediates, which lack the typical biaryl linkage and a tryptophan residue at the C-terminus. In an antiSMASH cluster search for Non-ribosomal perptide synthase (NRPS) BGCs, those coding for the productive pathway of daptomycin and arylomycin could be assigned to the produced antibiotics. The production of another known natural product [27], the *Pseudomonas*-active peptidylnucleoside napsamycin (Table 1) [29], was suggested by genome analysis and confirmed by the molecular network. Higher molecular weight variants seemed to be produced by *S. roseosporus* as well. Before it was unknown that *S. roseosporus* was able to produce napsamycins at all. The focus of this study lay on stenothricin, a barely characterized antibiotic that was discovered in 1974. So far, four analogs of stenothricin were known [30] and could be detected in the metabolome of *S. roseosporus*. A corresponding BGC, which matches the metabolomic information, could be identified in an antiSMASH analysis. Additionally, the existence of analogs of stenothricin were indicated by the molecular network (Table 1). These analogs differ in the length of the lipid side chains and amino acid substitution. Also, hydrolysed and glycosylated products could be identified. Liu et al. verified activity of the extracted stenothricin variant mixture against Gram-negative and Gram-positive bacteria [27].

This study demonstrates that it is worth investigating old strains with new methods, such as integrated genomic and metabolomic screening. As shown here, such an approach can result in an extended production profile of the known strain as well as point out bioactivities which have not been observed before.

In a study of Duncan et al. [24], 30 isolates of *Salinispora* strains were examined via combined genomic and metabolomic screening, also in order to detect new analogs. The species of the strains were identified as *Salinispora arenicola, Salinispora tropica* and *Salinispora pacifica.* Like *Streptomyces, Salinispora* belongs to the order of actinomycetes [32]. For several identified BGCs in the genome of the strains, no corresponding compound could be associated indicating that these clusters are not expressed under the cultured conditions or that the product is not detectable with the applied method. The raw extracts of the cultures were submitted to HR-MS/MS and a molecular network was calculated from the resulting ions. To identify the microbial products the MS-spectra were compared to a library of standard *Salinispora* biosynthetic products [24]. Besides the known compounds cyclomarin A [33] and D [34] (Table 1), the existence of a methylated, a demethylated and a hydrated analog is implied by MS-data. Also, a hydroxylated, dehydrogenated and a methylated version [24] of arenicolide A [35] could be detected (Table 1). Eight of the detected compounds could be linked to *Salinispora*-BGCs, among them the so far unidentified BGC of arenicolide A. The BGC could be associated with the production of arenicolide A as, on the one hand, this BGC was present exclusively in arenicolide producing strains and, on the other hand, the annotated ketosynthase-domains correspond to the sequence of the enzyme required for arenicolide production. Another BGC could be linked bioinformatically to a compound newly identified in this study, named retimycin A, an analog of the quinomycins [24], which display antiproliferative activity [36] (Table 1). Here, also the singleness of the parent ion as well as of the BGC in one single strain indicated the match. Additionally, similarity to a BGC of a known quinomycin-like compound was observed with variations in an adenylation-domain and a domain coding for an oxygenase. The MS/MS analysis indicated the presence of an oxidized, methylated thioacetal-version of retimycin A. This functional group has not been observed before for quinomycins [24]. So far, no bioactivity testing has been performed on retimycin A.

One advantage of genomic and metabolomic screening over classical bioactivity-based screening is the possibility to detect inactive analogs. Paulus et al. [31] investigated the biosynthetic potential of strain MP131-18, which was sampled at a Norwegian fjord. 16S phylogeny showed highest similarity to *Streptomyces specialis* and *Streptomyces avicenniae*. Culture extracts displayed activity against *Bacillus subtilis* and *Pseudomonas putida*. Analysis with antiSMASH revealed 36 gene clusters. To identify the corresponding metabolites HR-LC-QTOF MS was performed followed by dereplication with the Dictionary of Natural Products (DNP) database. This way, the production of the bisindole pyrrole antibiotics lynamicins A to G [37] and spiroindimicins B and C [38] could be confirmed. Known biosynthetic genes from the production of bisindole pyrroles were identified in BGC 36, so the cluster could be associated with the production of these compounds. Additionally, analogs of the known secondary metabolites (named lynamicin H, spiroindimicin E and spiroindimicin F; Table 1) could be identified in the culture extract [31]. Besides these, the polyketides lagunapyrones A-C [39] accumulated. Genes coding for type I and type III Polyketidesynthase (PKS) were present in BGC 3. This indicated that this cluster is responsible for lagunapyrone-production, as lagunapyrone is a polyketide. As for this type III PKS high flexibility in the choice of the acyl-CoA unit was predicted. In the following, the production of two more lagunapyrones D and E (Table 1) with C2 and C5 acyl-chains, of which masses could be found in the culture extract, was indeed confirmed [31]. The new compounds did not display antimicrobial activity with the exception of spiroindomicin B, which showed moderate activity against *Bacillus subtilis* [31]. Due to their lack of bioactivity, the analogs would not have been detected by classical activity-based screening of HPLC-fractions. 

All three studies presented in this section demonstrate that integrated genomic and metabolomic screening represents a useful tool for the discovery of unknown analogs of known compound classes and how the linkage between BGC and parent ion can be established.

### 2.2. Exploring the Productive Spectrum

Integrated genomic and metabolomic screening can be used to explore the productive capability of strains and prioritize those strains, which display the highest amount of uncharacterized genomic and metabolomic entities. 

Ishaque et al. [40] investigated the crude extract of a novel *Streptomyces* isolate named *Streptomyces tendae* VITAKN. The culture extract showed quorum sensing inhibition (QSI), thus the group aimed to identify the compound responsible for this effect. Hence, the group performed a whole genome analysis with antiSMASH, in which 33 BGCs could be detected. Only nine of these clusters showed more than 75% similarity to those deposited in the antiSMASH database. The remaining clusters were suspected to code for the production of so far unknown chemical entities. The crude extract was examined via LC-HRMS and LC-HRMS/MS. This resulted in a molecular network consisting of 327 nodes of which four correlated to the spectra of cyclic dipeptides (2,5-diektopiperazines) [40]. Cyclic dipeptides act as LuxR-type activators or inhibitors and exhibit antiproliferative, antibiotic and anti-inflammatory activity [41]. The genes coding for the key enzymes for the formation of 2,5-diektopiperazines, CDPS [42], were identified in the genome. A comparison of the spectra with data from the GNPS-MassIVE database did not result in exact matches. This indicates that the wanted compound has not been characterized before [40].

Nevertheless, the compound with QSI activity could not be identified via this combined genome and metabolomic screening approach. But as no corresponding MS-data for the detected parent ions could be found, an uncharacterized compound with QSI-activity is likely produced by *S. tendae* VITAKN. The existence of unexplored chemical entities also predestines the metabolome of *S. tendae* VITAKN as an object of further investigation [40].

A detailed screening of both, genome and metabolome, can not only help to estimate the amount of unknown natural products but also to elucidate the full biosynthetic potential of putative producer strains. *Streptomyces clavuligerus, Streptomyces jumonjinensis,* and *Streptomyces katsurahamanus* [23] are all known to produce the β-lactamase inhibitor clavulanic acid [43]. The following study by AbuSara et al. [23] aimed to examine if the three species produce other secondary metabolites in common. Therefore, a comparative analysis of the metabolome as well as of the genome was performed. Via LC-MS and LC-MS/MS analysis, it was observed that all three species produce desferrioxamines [44] and ectoine [45], which are very common in streptomycetes as these metabolites are required for general cell functions. The antibiotics holomycin [46] and thiolutin [47] are exclusively produced by *S. clavuligerus* [23]. In this study, the production of the antiproliferative nucleoside pentostatin [48] could be reported for the first time in *S. clavuligerus*, though the corresponding BCG had already been discovered in this strain. However, no production or BGC of pentostatin could be detected in *S. jumonjinensis* or *S. katsurahamanus* [23]. Naringenin is a flavonoid previously only known from plants [49] but was found here to be produced in all three *Streptomyces* species. The same is true for the plant-associated monoterpenes carveol and cuminyl alcohol. The terpene hydroxyvalerenic acid could be found in metabolome of *S. clavuligerus* exclusively. To elucidate the corresponding BGCs, an analysis with antiSMASH was performed. This way, 49 BGCs could be detected in the genomes of *S. jumonjinensis* and *S. katsurahamanus,* of which 44 could be associated with known clusters. Terpene like BGCs were observed in all three species, which could be the corresponding BGCs to the above-mentioned plant-derived metabolites. Besides clavulanic acid, *S. clavuligerus* is also able to produce its analog 5S clavam [23], which displays no inhibition of β-lactamases due to the inversed stereochemistry, and another unknown paralog of clavulanic acid [50]. While *S. jumonjinensis* and *S. katsurahamanus* are producers of clavulanic acid as well, no production of 5S clavam or the paralog could be detected. This is reflected in the genomes of the producer strains, which lack the according BGCs [23]. All three species are capable of producing cephalosporin C [51], which is linked to the production of clavulanic acid in *S. clavuligerus* [52]. The corresponding BGCs of *S. jumonjinensis* and *S. katsurahamanus* lack one gene, *blp*, of the cephalosporin-BGC in contrast to *S. clavuligerus*, which indicates that this gene is not essential for the production of cephalosporin C [23].

Though the group performed both, metabolomic and genomic analysis, still little connections could be drawn between the detected metabolites and the BGCs. Many nodes of the metabolomic clusters are unrelated to known BGCs, which reveals potential for further investigation [23]. However, the two methods complemented each other to create a more complete picture of the productive capability of the underexplored strains *S. jumonjinensis* and *S. katsurahamanus.*

Handayani et al. [53] aimed to explore the biosynthetic capabilities of *Streptomyces* isolates from rarely explored habitats in Indonesian terrestrial and marine environments. Firstly, 422 isolates were prioritized according to their bioactivity against a board of test microbes. Nine isolates were selected for integrated genomic and metabolomic screening, using antiSMASH and GNPS, respectively. Alongside metabolites required for basic cell functions (like desferrioxamines or spore pigments) several known compounds (ferrioxamine D1, naphthyridinomycin A, amicetin, antimycin A2, echinoserine, echinomycin, tirandamycin A, staurosporine) could be detected and associated with corresponding BGCs. The ion cluster of ECO-0501 could be detected but no corresponding BGC could be identified. In addition to these characterized compounds, four ion clusters were observed, to which no known compound could be associated. One of these clusters was probably attributed to peptides. The authors compared the BGCs of the three isolates, in which’s metabolome the peptidic ion clusters were observed, and identified two BGCs uniquely present in these three isolates. These BGCs presumably code for peptidic compounds, as they were identified as a bacteriocin and a NRPS/ectoine/butyrolactone/other/T1PKS gene cluster. Two more unidentifiable ion clusters were detected, for which a number of BGCs could code so that matching was not yet possible. The detected substances’ structures have not been elucidated yet [53].

By assigning the identifiable ion clusters and BGCs, the authors could prioritize strains and within the strains the ion clusters and BGCs, which probably code for new substances, for further research. The investigated strains displayed some unknown ion clusters and even more unknown BGCs, which could not be associated with the production of any known compounds. With these findings, the authors proved their point that underexplored habitats harbor untapped biosynthetic potential and demonstrated that an integrated genomic and metabolomic screening approach eases dereplication and prioritization.

Integrated genomic and metabolomic screening can also be applied the other way around, not to detect a new compound in a known strain but to find novel producers of known metabolites. In a study of Gosse et al. [54], the microbial biodiversity of the Iron Curtain Cave, Canada should be explored. Among the isolates, a strain displaying antimicrobial activity against multi-resistant *Escherichia coli* and non-resistant *Staphylococcus aureus* was selected for whole genome sequencing, followed by comparative genome analysis for taxonomic classification, which revealed high sequence identity to *Streptomyces lavendulae*. The investigated strain was accordingly named ‘*Streptomyces*’ sp. ICC1. For general cluster investigation, an antiSMASH analysis was performed. Additionally, the BAGEL4 algorithm was deployed. All in all, 37 gene clusters could be identified, the majority of which represent terpene clusters. The metabolome of ICC1 was investigated via UHPLC-ESI-HRMS followed by GNPS clustering. Nodes for several dipeptides were detected but no corresponding BGCs (NRPS or a cyclodipeptide synthase containing cluster) could be identified. On the other hand, a BGC for the siderophore coelichelin could be identified with 100% similarity via antiSMASH analysis, though the metabolite was not present in the culture extract. But then, siderophores are mainly produced under iron limiting conditions, while culture conditions for strains from an iron ore cave are maintained rich in iron. Additionally, some antibacterial compounds could be identified in the culture extract: 2′, 5′– dimethoxyflavone and nordentatin were detected by GNPS and a corresponding type III PKS cluster coding for both compounds could be identified via antiSMASH analysis [54]. Also, the antitumor [55] terpene diazepinomicin, which was only known to be produced by *Micromonospora* sp. DPJ12 so far, could be identified in the culture extract. The core genes for the key enzymes for the production of this compound, DkpF, DkpD, and DkpE, which form one of the two main building blocks of diazepinomicin [56], are present in one of the terpene clusters detected with antiSMASH [54].

Though no new antibacterial compounds could be detected, a new producer of the known antitumor agent diazepinomicin was identified. Additionally, the productive capabilities of the investigated strain indicate a high metabolomic potential, which should be explored further by established methods (fractioning culture extracts and prioritization according to their bioactivity). The study also demonstrates, that it is worth investigating both, genome and metabolome, on the one hand, to detect the potential for the production of secondary metabolites, even if they are not actually produced, and on the other hand, to detect metabolites even if the cluster has not been characterized so far.

### 2.3. Elucidation of Biosynthesis

Not only can new compounds be detected via combined genomic and metabolomic screening, it is also possible to identify their biosynthetic origin. For a study by Paulo et al. [57], *Streptomyces* sp. CBMAI 2042 was isolated from *Citrus sinensis* branches. This strain inhibited growth of *Bacillus megaterium, Staphylococcus aureus* and *Candida albicans* and suppressed the proliferation of *Citrus* pathogens. Whole genome sequencing followed by an analysis with antiSMASH revealed 35 BGCs, among them the NRPS-cluster for the depsipeptide valinomycin. Valinomycin has proven antibacterial [58], antiviral [59] and antiproliferative [60] properties. Via UHPLC-QTOF-MS/MS the metabolite itself and its ammonium adduct were detected and the identities verified via matching with the GNPS database. Additionally, the bioinformatic tools DEREPLICATOR and VarQuest, which are specialized in the detection of peptide natural products, were used. Both, valinomycin and its ammonium adduct, appeared as distinct nodes in the molecular network. Besides valinomycin, *Streptomyces* sp. CBMAI 2042 was able to produce the cyclic depsipeptide montanastatin [57], which also displays antiproliferative activity [58]. Though montanastatin has been known before, the biosynthetic origin has never been elucidated. But according to its structure, montanastatin could stem from the same cluster as valinomycin. Thus, the authors expressed the cluster for valinomycin in *S. coelicolor* as heterologous host and performed a metabolomic analysis. Montanastatin was detected as well as valinomycin and five so far unknown valinomycin-analogs (Table 1). This demonstrates, that all mentioned metabolites originate from the same NRPS-cluster.

Here, metabolomic and genomic approaches were combined in order to elucidate the common biosynthetic origin of valinomycin and montanastatin. Nevertheless, heterologous expression for confirmation remained indispensable.

Liu et al. [25] also aimed to elucidate biosynthetic details by using combinatorial genetic and metabolomic screening to determine the absolute configuration of the stereochemical centers of the newly isolated strecacansamycins A, B and C (Table 1), which are produced by *Streptomyces cacaoi* subsp. *asoensis* H2S5. Strecacansamycins belong to the class of aliphatically bridged aromatic ansamycins [61]. In activity tests in vivo against PC-3, HepG2, and U87-MG cells, respectively, the isolated analogs displayed antiproliferative properties [25]. LC-HR-ESI-MS data of the culture extract were evaluated with GNPS. This way, nodes for ansamycin-analogs were detected. MS data, however, give no information about the absolute stereochemistry of a molecule. So, Liu et al. used the genetic information to reconstruct the production pipeline and derive which stereochemistry would be provided by the modules. For this purpose, the whole genome was sequenced and analyzed with antiSMASH. The analysis revealed 31 BGCs. A type I PKS-NRPS hybrid cluster is probably responsible for the production of strecacansamycins. PKSs and NRPSs are composed of several biosynthetic units called modules, which contain a set of catalytically active domains. The type I PKS-NRPS hybrid cluster contains acyltransferase-domains in module 4, which are stereoselective for *S*-methylmalonyl-CoA, but stereoinversion occurs in the subsequent condensation reaction catalyzed by a ketosynthase-domain so the final configuration at C-12 is *R*. The configuration of the methoxy or hydroxyl-groups at C-3, C-11 and C-13 could be determined as *R, S* and *R,* based on the direction of the hydride-addition at the ketoreductase-domains. One ketoreductase-domain type is also responsible for the formation of *cis* or *trans* double bonds depending on the direction of the reduced hydroxy-group. This way, it could be deduced that the double bonds at C-5, C-7, and C-9 exhibit *trans*- and those at C-15 *cis*-configuration [25].

The study demonstrates the usefulness of integrated genomic and metabolomic screening for structure elucidation beyond the scope of mass spectrometry, especially concerning stereochemistry. This is of special relevance considering the stereospecificity of most mechanisms of action.

## 3. Conclusions

The listed examples demonstrate that integrated genomic and metabolomic screening has already been successfully applied for various purposes: The strategy has proven useful for comparative genomic and metabolomic studies as to explore the full biosynthetic potential of known or newly isolated *Actinomyces* strains and to detect similarities in the production profile of distinct strains [23,40,53]. This way, new producers of known compounds could be found [24,54]. Based on this overview of their productive capabilities, strains could be prioritized according to the presence of unidentifiable clusters [40]. Also, bioactive as well as inactive analogs of known compounds have been found [27,31]. Therefore, integrated genomic and metabolomic screening also facilitates the elucidation of biosynthesis [25,57].

Nevertheless, integrated genomic and metabolomic screening should not be overestimated, for it will not enable the discovery of new scaffolds, but just support this objective by easing the process of dereplication. No completely new lead structures could be discovered with the method so far, due to the fact that the compound and BGC identification is based on a pattern-based search, which relies on acquired data about known compounds and BGCs. For the identification of new compound-classes laborious bioactivity-guided screening, genetic engineering, and fractionation of culture extracts, as well as heterologous expression will still be required.

In conclusion, integrated genomic and metabolomic screening is a useful tool for simplified dereplication and as such will help to accelerate drug discovery through identification of new strains and analogs of known bioactive compounds.

## Figures and Tables

**Table 1:** List of newly discovered compounds from actinomycetes via combined genomic and metabolomic screening.

Compound	Strain	Activity	Reference
napsamycin analogs stenothricin analogs	*Streptomyces roseosporus*	antibiotic	[27]
**spiroindimicins E and F** **lagunapyrones D and E**	*Streptomyces* sp. MP131-18	none	[31]
strecacansamycin A, B, C	*Streptomyces cacaoi* subsp. *asoensis*	antiproliferative	[25]
valinomycin derivatives	*Streptomyces* sp. CBMAI 2042	antibiotic	[26]
**cyclomarin analogs** **arenicolide analogs** **retimycin A**	*Salinispora* sp.	suggested antiproliferative	[24]

## Data Availability

Data sharing not applicable.
